# Molecular mechanisms underpinning cognitive reserve and resilience

**DOI:** 10.3389/fnagi.2026.1957901

**Published:** 2026-10-09

**Authors:** Amber Chen, Sreenithi Bhuvaneshwaran, Yash Akkara, Thomas Burke, Robert Perneczky, Magdalena Sastre

**Affiliations:** 1 Division of Neuroscience, Department of Brain Sciences, Imperial College London, London, United Kingdom; 2 Department of Psychiatry and Psychotherapy, LMU Hospital, Ludwig-Maximilians University Munich, Munich, Germany; 3 German Centre for Neurodegenerative Diseases (DZNE) Munich, Munich, Germany

**Keywords:** aging, Alzheimer’s disease, cognitive reserve, lifestyle, resilience, synapses

## Abstract

The concept of cognitive reserve (CR) and resilience originates from clinicopathological observations that amyloid-β deposition, tau pathology and neurodegeneration do not uniformly translate into clinical dementia, highlighting inter-individual differences in the brain’s capacity to tolerate pathology. Although CR and resilience lack a single accepted definition, they broadly describe the ability to maintain cognition in the presence of age-related or pathological brain changes. This review synthesises current molecular, cellular, and brain network mechanisms of cognitive reserve and critically examines key controversies. We also identify convergent biological pathways across multiple levels of organisation. Available evidence implicates that CR is not attributable to a single factor but reflects interactions among environmental exposures, genetic background, epigenetic regulation, cellular homeostasis, glial function, and large-scale brain network organisation. Lifestyle factors are associated with reduced pathology accumulation, preserved brain structure, and maintenance of cognition despite pathology, depending on the mechanism and outcome considered and are understood to exert their effects through epigenetic modulation of neuroplasticity, neurotrophic signalling, metabolic regulation, and inflammatory pathways. At the cellular level, preservation of dendritic spine integrity, synaptic signalling balance, mitochondrial energetic flexibility, and adult hippocampal neurogenesis supports efficient circuit function under pathological stress. Glial cells play central roles in mediating adaptive plasticity, neuroinflammatory control, and metabolic support, with dysregulation accelerating cognitive decline. Collectively, these findings frame CR as an emergent property of coordinated biological processes operating across molecular, cellular, and network scales, with important implications for developing reserve-enhancing therapeutic and lifestyle-based interventions that complement traditional pathology-focused approaches.

## Graphical abstract

**Figure:**
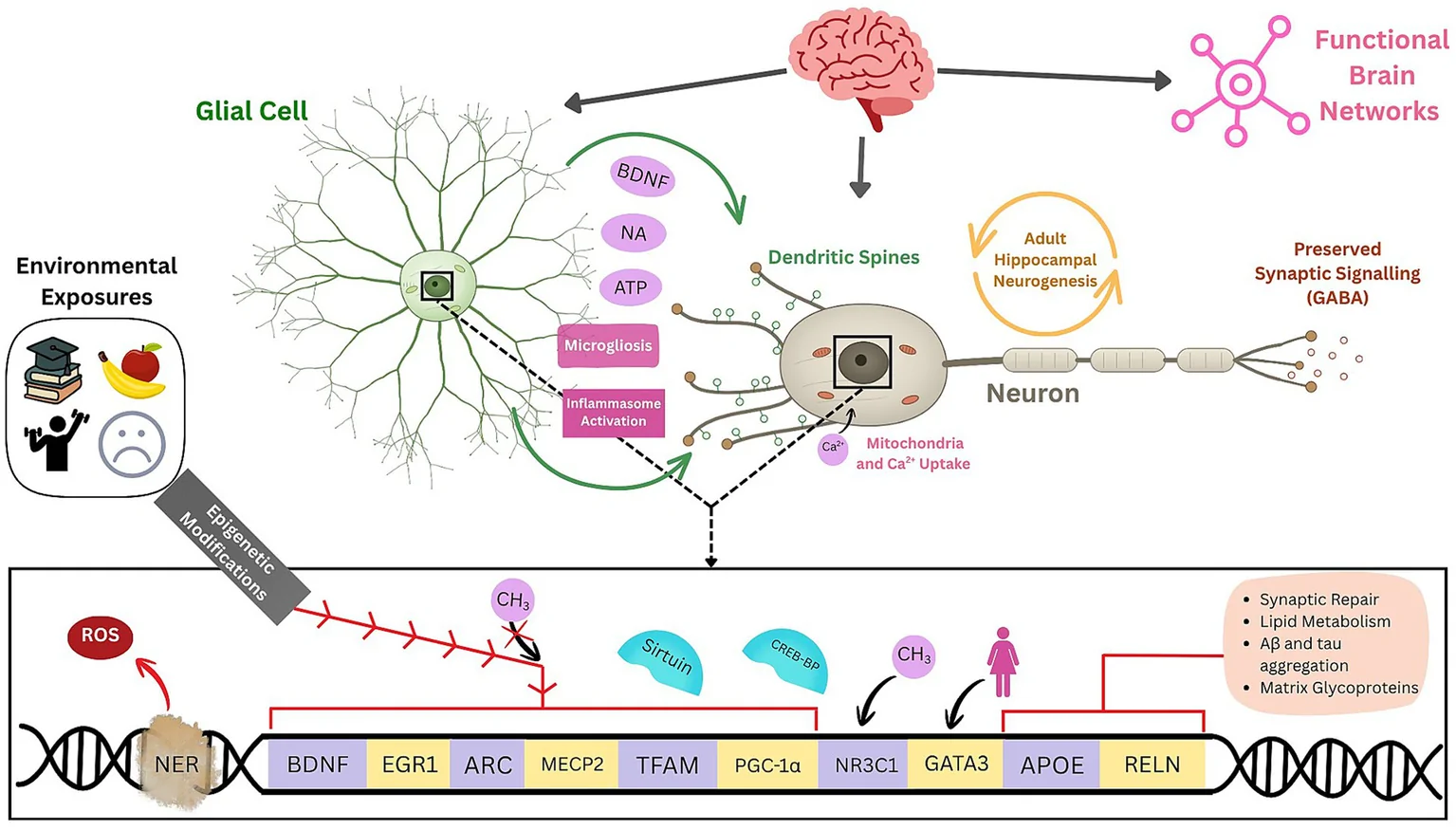
Multi-level schematic of the mechanisms underlying cognitive reserve (CR). Environmental exposures including education, diet, physical activity and stress act on the genome through epigenetic modifications, regulating genes involved in synaptic plasticity, mitochondrial function, stress signalling and lipid and matrix handling. Because this programme is shared by neurons and glia, it sets the balance between trophic gliotransmission that maintains dendritic spines and reactive inflammation that erodes them. Glial release of BDNF, noradrenaline and ATP support neuronal plasticity and metabolic demand, while microgliosis and inflammasome activation shift this support towards synaptic loss. Preserved spine stability, mitochondrial flexibility, adult hippocampal neurogenesis and sustained inhibitory signalling together support efficient synaptic transmission, which scales up to the resilient functional brain networks that constitute CR.

## 1 Introduction

The concept of cognitive reserve (CR) and resilience emerged from observations such as those from the longitudinal Nun Study (Clarke et al., 2025), in which some individuals with Alzheimer’s disease (AD) pathology maintain relatively preserved cognitive function. Post-mortem and in vivo studies have consistently shown that amyloid-β deposition, tau pathology, and neurodegeneration do not uniformly translate into clinical dementia (Stern, 2012). This suggests that inter-individual differences in the brain’s capacity to tolerate pathology influence cognitive outcomes, forming the basis of the resilience framework. In this line, recent research in cognitively healthy centenarians in the 100-plus Study has demonstrated that some are able to maintain cognitive health despite substantial AD pathology, whereas others showed very limited pathology. Particularly, these observations suggest that centenarians preserve cognition either through resistance to Aβ accumulation or resilience to Aβ-induced p-tau pathology (Hulsman et al., 2026). The essential distinction is that resistance concerns whether pathology develops, whereas resilience concerns the cognitive consequences once pathology is present.

Clinically, the threshold and decompensation model suggests that reserve shifts the relationship between pathology and symptoms rather than slowing pathological progression (Pettigrew et al., 2020). Individuals with greater reserve can tolerate higher pathological burden before symptoms emerge (Li et al., 2021). However, once compensatory capacity is exhausted, decline may be more rapid because clinical expression occurs at a more advanced disease stage (Soldan et al., 2017). This process reflects an initial adaptive response involving compensatory neural activity and other protective mechanisms, which eventually become overwhelmed, leading to neuroinflammation, metabolic dysfunction, network breakdown, and clinical deterioration (Stern, 2012; Hillary and Grafman, 2017; Penalba-Sánchez et al., 2022; Liu et al., 2025).

Although the terms are widely used, CR and resilience lack a single, universally accepted definition and are often used interchangeably across the literature. To avoid conceptual ambiguity, we distinguish mechanisms that influence the accumulation of brain ageing or disease, from mechanisms that modify the cognitive consequences of these changes. Brain maintenance refers to reduced or delayed age-related brain change, whereas resistance refers more specifically to reduced accumulation or spread of neuropathology. Brain reserve denotes quantitative or structural brain features that increase the amount of damage that can be sustained before clinical impairment becomes apparent. CR refers to active adaptability of cognitive and neural processes, including neural efficiency, capacity, flexibility and compensatory network recruitment (Stern et al., 2023). Here, resilience is used as an overarching clinical phenotype, denoting better-than-expected cognitive function given the observed burden of pathology or neurodegeneration. These constructs are related but not interchangeable, and individual biological mechanisms may contribute to more than one construct. A given pathway may reduce pathology accumulation, preserve structural substrate, and facilitate functional adaptation. Its classification therefore depends on the level and outcome being examined rather than on the molecular pathway alone (Stern et al., 2020) (see Supplementary Table S1).

CR research has been driven by the belief that identifying its underlying biological mechanisms could lead to novel therapeutic targets (Ma and Pop, 2024). Various lifestyle and environmental factors, such as education, occupation, activity, social engagement, and cognitive stimulation, have been associated with CR. However, these are likely proxies for underlying biological processes, such as brain-derived neurotrophic factor (BDNF) release, which is modified by diet and exercise and enhances synaptic density, neuronal survival, and neurogenesis (Alqahtani et al., 2025). Research has increasingly shifted towards identifying the molecular, cellular, and systems-level pathways that enable neural compensation for neurodegenerative pathology. Advances in neuroimaging, genomics, epigenomics, proteomics, and single-cell transcriptomics have provided new insight into the biological basis of CR.

Despite these advancements, the field remains fragmented, with findings often examined in isolation and occasionally producing conflicting results. Perhaps the biggest controversy is that CR is a theoretical construct rather than a directly measurable biological entity. Moreover, the molecular mechanisms that drive CR remain incompletely understood, limiting translation of these findings into effective disease-modifying therapies.

In this review, we examine how environmental and genetic factors influence resistance and brain maintenance, how cellular and structural mechanisms contribute to brain reserve, and how synaptic and network adaptations generate cognitive reserve and ultimately cognitive resilience.

A central proposition of this review is that cognitive reserve is not a property of any single biological level but an emergent phenomenon arising from interactions across levels. Genetic and environmental factors set the operating range of cellular maintenance pathways, which influence synaptic and circuit efficiency and ultimately determine network-level adaptability. This framework predicts that biomarkers measured across levels will explain resilient cognitive performance better than markers derived from any single scale alone.

A detailed mechanistic understanding of CR could offer a pathway towards developing novel therapeutic strategies aimed at preserving cognition.

## 2 Environmental exposures and epigenetic regulation on CR

Lifetime environmental exposures contribute to cognitive reserve (CR) by shaping cognitive outcomes through modifiable factors, unlike genetic susceptibility, which is largely fixed. Epigenetic regulation provides a plausible mechanism linking environmental experiences to CR, as exposure-dependent changes in gene expression and neural networks have been observed in pathways related to learning and memory (Koulouri and Zannas, 2024). While most research has focused on the relationship between epigenetic modifications and neurodegenerative burden or structural brain reserve, growing evidence suggests that epigenetic processes may also support the functional adaptations characteristic of CR.

### 2.1 Environmental enrichment

Cognitively stimulating lifestyles, including higher educational and occupational attainment, are consistently associated with preserved cognition despite ageing and neurodegeneration (Melendez et al., 2026). Because of this relationship, education is often used as a proxy measure of CR (Stern et al., 1999). In rodent models, environmental enrichment enhances learning, memory and hippocampal neurogenesis (Khalil, 2024), although the persistence of these effects depends on the duration and timing of exposure. However, CR cannot be inferred from education alone. It is most appropriately operationalised as inter-individual variation in cognition that remains after accounting for demographic factors and objectively measured brain ageing or pathology, or as moderation of the relationship between pathology and longitudinal cognitive change.

Environmental enrichment induces widespread epigenetic changes within the hippocampus, including DNA hypomethylation and increased expression of neuroplasticity-related genes such as *BDNF*, *EGR1* (Early Growth Response Protein 1) and *ARC* (Activity-Regulated Cytoskeleton-Associated Protein) (Puang et al., 2020; Costa et al., 2023). Age-related methylation changes at the *MECP2* (Methyl-CpG-Binding Protein 2) locus can also be partially reversed (Huang et al., 2021; Zocher et al., 2021), supporting theories of “epigenetic rejuvenation,” whereby stimulating environments mitigate aspects of biological ageing. However, these modifications persist for only a limited period after removal from the enriched environment (Zocher et al., 2020), suggesting that sustained cognitive engagement may be necessary to maintain long-term benefits.

### 2.2 Lifestyle exposures

Lifestyle factors such as diet, physical activity, and psychosocial stress may influence CR through epigenetic mechanisms. Dietary factors can regulate DNA methylation and histone modifications in genes involved in synaptic function, neuroinflammation, oxidative stress, and neuronal plasticity (Li et al., 2024).

Caloric restriction is of particular interest because it activates epigenetic regulators, including Sirtuin-1 (SIRT1) and CREB-binding protein (CREB-BP), increasing chromatin accessibility and promoting expression of neuroplasticity-related genes such as *BDNF* (Koulouri and Zannas, 2024; Alkurd et al., 2024). Conversely, high-fat diets induce maladaptive epigenetic changes associated with cognitive decline. However, the causal relationship between diet, *BDNF* signalling and CR remains unclear (Xie et al., 2023; Sequeira-Cordero et al., 2025).

Physical activity appears to promote CR through epigenetic regulation of neurotrophic, synaptic, and mitochondrial pathways, increasing expression of *BDNF*, *MEF2* (Myocyte Enhancing Factor 2), *PPAR-δ* (Peroxisome Proliferator-Activated Receptor-δ), *PGC-1α* (PPARγ Coactivator-1α) and (*TFAM* Mitochondrial Transcription Factor A), while also altering microRNA profiles involved in synaptic remodelling and neuronal metabolism (Benite-Ribeiro et al., 2023; Steinke et al., 2023).

In contrast, chronic psychosocial stress has been linked to increased dementia risk through epigenetic alterations of the glucocorticoid receptor gene *NR3C1*, (Nuclear Receptor Subfamily 3, Group C, Member 1), leading to disrupted cortisol regulation, impaired hippocampal neurogenesis, and poorer memory performance (Gassen et al., 2017).

Together, these findings suggest that lifestyle exposures contribute to CR through epigenetic pathways, although causal relationships remain to be fully established.

## 3 Genetic influences on CR

Rising evidence supports a polygenic model for the genetic influence on mechanisms underlying CR and resilience. Several population-level genome-wide association studies (GWAS) highlight gene sets mapping to biological pathways with potential mechanistic relevance. The effect of AD-risk genes and specific mutations is also relevant to understanding the association between gene expression and resilience.

Large-scale GWAS demonstrate that resilience-associated variants show minimal overlap with clinical AD risk, including weak or absent associations with Apolipoprotein (APOE) ε4. This dissociation supports a dual-pathway idea, whereby AD risk genes influence pathology accumulation, while resilience genes, such as the APO ε3 Christchurch mutation, modify the cognitive consequences of pathology. Table 1 details key genetic loci implicated in CR and resilience.

**Table 1:** Summary of genetic loci or pathways associated with cognitive reserve/resilience, grouped by mechanism, key variants and type of evidence.

Mechanism contributing to cognitive reserve/resilience	Locus/pathway	Function	Key variant	Study type	Reference(s)
DNA repair and oxidative stress	Nucleotide excision repair	DNA repair, reactive oxygen species removal	–	GWAS	Ko et al. (2022)
	Metallothionein	Reactive oxygen species removal	–	Transcriptomic	de Vries et al. (2024)
Lipid metabolism and protein trafficking	*ATP8B1*	Bile acid metabolism, prefrontal cortex methylation	–	GWAS	Dumitrescu et al. (2020)
	*APoE*	Lipid metabolism, synapse plasticity, membrane repair	–	Cohort studies	Pettigrew et al. (2023)
			*APOE ε3* Christchurch	Case report/animal model	Arboleda-Velasquez et al. (2019) and Naguib et al. (2025)
Neuroinflammation	*PLCG2*	Modifies neuroinflammation, microglial phagocytosis	*P522R*	GWAS	Tesi et al. (2024)
	*GATA* binding protein 3	Neuronal survival and development, microglial gene expression	–	GWAS	Eissman et al. (2022)
Synaptic signalling and plasticity	Tumour growth factor-β	Synaptic plasticity and neuronal survival	–	GWAS	Phillips et al. (2025)
	*APP*	Synaptic plasticity, neurite growth	–	TWAS/GWAS	Mustafa et al. (2025)
	*PICALM*	Synapse function, endothelial function	–	Candidate gene	Ponomareva et al. (2020)
			rs3851179	GWAS	Eissman et al. (2022)
	*RAB10*	Synaptic vesicle trafficking	rs142787485	Pedigree WGS	Ridge et al. (2017)
	*CLU*	Synaptic maintenance	rs11136000	Multi-omics	Ma and Qiu (2022)
	*BDNF*	Synaptic plasticity, neurotrophic support	Val66Met	Candidate gene	Ward et al. (2015)
Neural network organisation and synapse connectivity	*NPTX2*	Synaptic connectivity, network integrity marker	–	Proteomic	Oh et al. (2025)
	*RELN*	Neuronal migration, network organisation	–	GWAS	Kramer et al. (2011)
			*RELN-COLBOS*	Case report	Lopera et al. (2023)
Endothelial and mitochondrial metabolism	Endothelial and mitochondrial loci	Blood-brain-barrier integrity; neuronal energy supply	–	PRS	Xu et al. (2024)

APP: Amyloid Precursor protein; ATP8B1: ATPase phospholipid transporting 8B1; CLU: Clusterin; PLCG2: Phospholipase C gamma 2; PICALM: Phosphatidylinositol binding clathrin assembly protein; RAB10: Ras-related protein Rab-10; BDNF: Brain-derived neurotrophic factor; NPTX2: Neuronal pentraxin 2; RELN: Reelin; GWAS: genome-wide association study, TWAS: transcriptome-wide association study, WGS: whole genome sequencing, PRS: polygenic risk score; - represents a lack of identified key variant.

Collectively, these analyses support the roles of DNA repair, oxidative stress regulation, synaptic and neuronal maintenance, and glial-neuronal signalling in CR and resilience, concepts revisited in subsequent sections.

## 4 Intracellular and neuronal mechanisms as surrogate drivers of reserve and mesoscale synaptic/circuit dynamics

CR is supported by the ability of neurons to maintain synaptic integrity, energetic homeostasis and neurogenesis despite ageing and neuropathology. Cellular and intracellular processes that preserve these functions may therefore act as surrogate drivers of CR, enabling neural circuits to sustain cognition under adverse conditions (see summary in Figure 1) (Anacker and Hen, 2017; Chang and Hen, 2024; Taddei and Duff, 2025).

**Figure 1:**
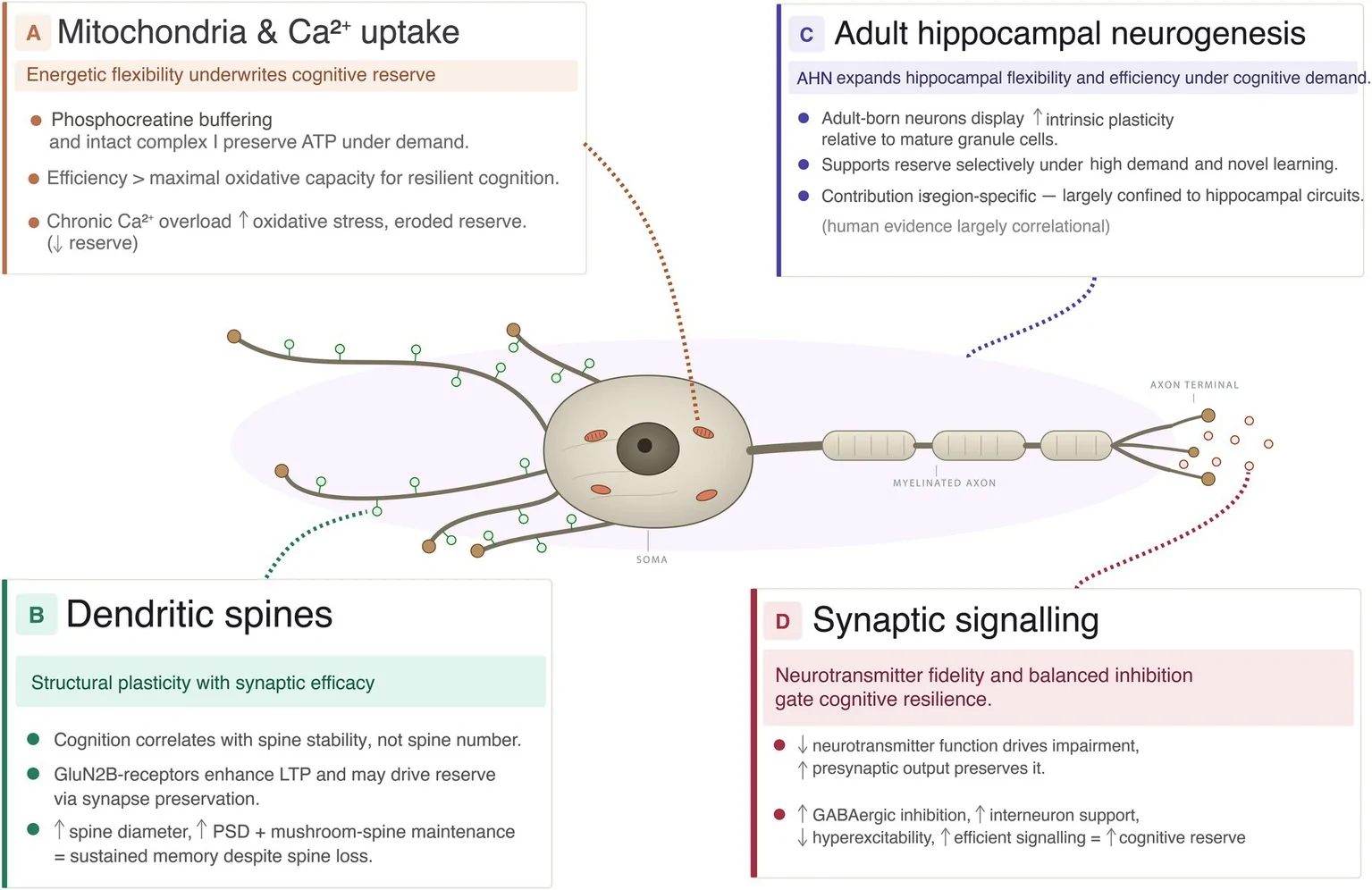
Schematic overview of how coordinated neuronal processes support cognitive reserve (CR) under ageing and disease. (A) Mitochondrial energetic flexibility and regulated Ca2+ handling enable neurons to meet metabolic demands without accumulating damage. (B) Preserved dendritic spine stability, (C) adult hippocampal neurogenesis, and (D) efficient synaptic signalling sustain memory despite synaptic loss, while enhanced inhibitory control limits network hyperexcitability. Adult hippocampal neurogenesis further contributes by increasing circuit flexibility under high cognitive demand, highlighting CR as an emergent property of interacting intracellular mechanisms. (www.canva.com).

### 4.1 Mitochondrial energetics and Ca^2^^+^ regulation

The capacity of neurons to sustain compensation also depends on mitochondrial function. Rather than absolute oxidative capacity, CR appears to be associated with energetic flexibility, including efficient phosphocreatine buffering and preserved mitochondrial complex I activity (Mahapatra et al., 2023; Shatalina et al., 2024; Lopez et al., 2025). These processes enable neurons to meet fluctuating energy demands while limiting cumulative cellular damage.

Controlled transfer of Ca^2+^ between synapses and mitochondria supports synaptic plasticity, whereas chronic Ca^2+^ overload promotes oxidative stress, mitochondrial dysfunction and synaptic failure (Arnsten et al., 2025; Ebrahimi et al., 2025). Regulation of endoplasmic reticulum-mitochondria coupling may therefore represent an intracellular reserve mechanism that preserves neuronal function during stress. However, direct evidence linking mitochondrial Ca^2+^ regulation to CR in humans remains limited, with most findings derived from animal and in vitro studies (Popugaeva et al., 2017).

### 4.2 Adult hippocampal neurogenesis

Adult hippocampal neurogenesis (AHN), the generation of new granule neurons in the adult dentate gyrus, may support CR by improving the efficiency of hippocampal information processing rather than replacing lost neurons (Anacker and Hen, 2017; Chang and Hen, 2024). Newly generated neurons undergo a period of heightened excitability and plasticity that enhances memory encoding and discrimination (Akers et al., 2014; Frechou et al., 2024).

By improving pattern separation and reducing interference between memories, AHN may enable hippocampal networks to operate more efficiently under cognitive stress, effectively expanding reserve capacity (Danielson et al., 2016; McAvoy et al., 2016). However, its contribution is constrained by the marked decline of neurogenesis with ageing and the requirement for successful integration of new neurons into existing circuits. Furthermore, much of the evidence derives from murine studies, and the extent of AHN in humans remains debated (Boldrini et al., 2018; Sorrells et al., 2018).

Overall, these findings suggest that CR emerges from coordinated intracellular processes that preserve synaptic signalling, energetic adaptability and neurogenesis. Together, these complementary mechanisms provide a biologically plausible explanation for why some individuals maintain cognitive function despite substantial neuropathology.

### 4.3 Dendritic spines and synaptic signalling

Dendritic spines, the principal postsynaptic sites of excitatory transmission, play a key role in linking structural plasticity with synaptic efficacy. Their preservation may support CR by maintaining communication within neural networks (Guptarak et al., 2024; Bhembre et al., 2025). Across ageing, cognitive performance has been associated with spine morphology and stability, particularly the maintenance of GluN2B-containing receptors that facilitate long-term potentiation (Pegasiou et al., 2020; Guptarak et al., 2024). Emerging evidence also identifies synapse-related biomarkers, such as the YWHAG:NPTX2 ratio, as predictors of cognitive impairment independent of pathological burden, reinforcing synaptic integrity as a substrate of reserve (Oh et al., 2025).

Preservation of spine diameter, postsynaptic density and mature mushroom spines appears capable of sustaining memory performance despite overall spine loss, potentially accounting for compensatory processes observed in ageing and dementia (Aguilar-Hernández et al., 2025; Monroy et al., 2025). However, these effects are often region- and task-specific, and evidence in humans remains largely correlational.

The effectiveness of structural compensation depends on intracellular signalling. Individuals demonstrating resilience frequently exhibit preserved presynaptic function and inhibitory control, supported by GABAergic signalling and inhibitory interneuron activity (Morgan and Carlyle, 2024; van Dyck et al., 2024). These mechanisms may limit network hyperexcitability, maintain signal fidelity and reduce metabolic strain, thereby contributing to CR.

### 4.4 Mesoscale synaptic/circuit dynamics

Mesoscale circuit dynamics are critical regulators of CR, enabling adaptive neural reorganisation in response to pathology. CR depends on maintaining excitatory-inhibitory balance, with stable cortical excitatory and hippocampal inhibitory ensembles preventing amyloid-driven hyperconnectivity (Chen et al., 2026). Disruption of inhibitory signalling, including parvalbumin interneuron dysfunction and reduced GABA_A receptor α1 expression, promotes neuronal hyperactivity, whereas targeted activation of inhibitory microcircuits can reverse early cognitive deficits (Shu et al., 2023). Early Aβ-related circuit dysfunction is also reflected by interictal epileptiform spikes during low-arousal states, which may predict later memory decline (Shoob et al., 2023).

At the systems level, these adaptations extend across distributed functional brain networks.

### 4.5 Functional brain networks

CR relies on functional network integrity despite neuropathology. Stronger default mode network (DMN)-visual network coupling may compensate for hippocampal dysfunction and support cognition (Jiang et al., 2025). Reserve-related activity also involves inferior temporal and occipital regions, including the fusiform gyrus (Vockert et al., 2024). Dynamic connectivity studies further show that favourable risk profiles are associated with strongly connected and DMN-linked network states, whereas weaker connectivity patterns are linked to lower cognitive engagement and greater vascular or mood-related risk (Dautricourt et al., 2022). Together, these findings suggest that resilience depends on the brain's ability to maintain and redistribute connectivity across networks in response to pathology.

## 5 Glial cells

In addition to cellular mechanisms operating within neurons, glial cells are increasingly recognised as important mediators of CR and resilience. By regulating neural activity, metabolism, and inflammation, these cells influence the brain’s capacity to maintain cognition despite ageing and pathology.

### 5.1 Astrocytes

Astrocytes are increasingly recognised as key contributors to cognitive resilience, functioning as molecular and metabolic integrators rather than passive support cells. Supporting this view, a recent plasma proteomic study identified astrocyte ageing as the strongest cellular predictor of incident AD, with accelerated astrocyte ageing associated with greater disease risk (Ding et al., 2026).

Astrocytes support CR by maintaining synaptic signalling through neurotransmitter recycling, neurotrophic factor release, and Ca^2+^-mediated regulation of neural network activity (Zonca et al., 2025). They also act as metabolic buffers (Mason, 2017) and regulate cerebral blood flow via neurovascular coupling, suggesting that resilience may depend not only on neuronal capacity but also on astrocytic metabolic and vascular support (Attwell et al., 2010; Iadecola, 2017). However, the contribution of neurovascular function to CR remains relatively understudied.

### 5.2 Microglia

Microglia also play a key role in CR through both neuroprotective and immune-related functions. Adaptive microglial responses may promote resistance by limiting tau propagation (Jury-Garfe et al., 2024), supporting brain maintenance by reducing chronic inflammatory injury, and indirectly supporting cognitive resilience by preserving synaptic and circuit integrity. In addition to secreting anti-inflammatory cytokines and neurotrophic factors such as BDNF, microglia regulate synaptic pruning, refine neural circuits, and potentially enhance network efficiency (Schafer et al., 2012; Favuzzi et al., 2019; Li et al., 2023). However, excessive or chronic microglial activation is associated with greater amyloid and tau pathology and poorer cognition (Rossano et al., 2024).

Together, these findings suggest that adaptive microglial responses support CR, whereas chronic neuroinflammation undermines resilience.

### 5.3 Oligodendrocytes

Oligodendrocytes contribute to neural reserve by maintaining myelin integrity, processing speed and network integration (Nave, 2010). Although the causal role of demyelination in cognitive impairment remains uncertain, remyelination improves learning and memory in rodent models, suggesting that preserved myelin may enable more efficient cognitive processing (Pan et al., 2020; Steadman et al., 2020).

## 6 Discussion: therapeutics/translational perspectives

Despite increasing evidence linking these mechanisms to CR, several challenges limit their therapeutic translation. A major issue is the lack of standardised definitions and measurements of CR, which hinders reproducibility and cross-study comparisons (Stern et al., 2020). Furthermore, reliance on proxies such as education and occupation may introduce socioeconomic and cultural confounds (Melendez et al., 2026). The impact of reserve mechanisms is likely moderated by age, sex, socioeconomic factors and disease stage, variables that remain insufficiently addressed in current mechanistic studies. Another limitation is that most human evidence remains observational, whereas mechanistic support derives predominantly from experimental animal studies.

The field is also dominated by cross-sectional and correlational research. Longitudinal and interventional studies are needed to establish causality, particularly for potentially reversible mechanisms such as epigenetic regulation and glial modulation. Such work will clarify whether targeting these processes can enhance resilience and CR.

Future strategies may focus less on modifying pathology and more on preserving the compensatory mechanisms underlying CR (Zammit et al., 2024). Lifestyle interventions are promising, with exercise activating FNDC5/irisin and BDNF-TrkB signalling pathways that support hippocampal plasticity and cognitive resilience (Lourenco et al., 2019; Cheng et al., 2025). Dietary polyphenols similarly promote neurogenesis and reduce oxidative stress, while combined exercise and dietary interventions may further improve cognitive outcomes (Carrillo et al., 2025; Komulainen et al., 2021).

Emerging pharmacological therapies include TREM2 agonists, BDNF mimetics, APOE-targeted gene therapies, histone deacetylase inhibitors, NAD^+^ precursors, and GLP-1 receptor agonists (Jackson et al., 2024; Li et al., 2025; Numakawa and Kajihara, 2025; Qader et al., 2025). Circuit-level neuromodulation approaches, including transcranial magnetic stimulation, transcranial direct current stimulation, and sensory flicker stimulation, also show promise for enhancing synaptic plasticity and resilience (Adaikkan et al., 2019; Yu et al., 2019; Tang et al., 2021).

The most effective approaches will likely be multi-faceted, combining reserve-enhancing molecular, neuromodulatory and behavioural interventions. Future research should adopt multimodal and multi-omic approaches integrating molecular, genetic, epigenetic and neuroimaging markers of neural efficiency (Yang et al., 2025), including biomarkers of neurodegeneration and structural and functional measurements of network efficiency by imaging.

The framework generates several testable predictions. First, individuals with comparable amyloid and tau burden should differ in cognition according to multiscale reserve signatures combining genetic, glial, synaptic and network biomarkers. Second, reserve-enhancing lifestyle interventions should produce measurable changes in molecular and network markers before detectable cognitive effects emerge. Third, biomarkers of inhibitory network stability and synaptic integrity should predict resilience more strongly than pathology measures alone. So far, no biomarker has achieved broad acceptance. Finally, interventions targeting multiple levels simultaneously may produce larger effects than interventions focused on a single mechanism.

## 7 Conclusion

In conclusion, elucidating the mechanisms that underpin CR and resilience is essential for the development of targeted therapeutic strategies that may transform the current approach to neurodegenerative diseases, such as AD treatment. The factors discussed in this review should not be viewed as isolated processes but rather as components of an integrated mechanistic continuum, spanning genetic and environmental modifiers, molecular and cellular pathways, synaptic and network-level adaptations, and ultimately clinical phenotypes (Graphical Abstract). BDNF signalling illustrates a potential cross-level pathway whereby environmental exposures influence epigenetic regulation, synaptic plasticity, network efficiency, and ultimately resilient cognition. Importantly, although many mechanisms described here are related to AD, emerging CR-focused therapeutic approaches have the potential to extend beyond AD, offering novel avenues for promoting resilience and modifying disease trajectories across a broad range of neurological disorders. As our understanding of these interconnected mechanisms advances, harnessing CR may become a central strategy for preserving cognitive function and improving clinical outcomes in neurodegenerative disease.
