# Supplementary material for: Molecular mechanisms underpinning cognitive reserve and resilience
Source: Front Aging Neurosci. 2026 Oct 9;18:1957901. doi: 10.3389/fnagi.2026.1957901 (PMC13692622; doi:10.3389/fnagi.2026.1957901)
Supplement: Supplementary file 1 [file Table_1.docx]

**Supplementary Table 1: Biological mechanisms classified by predominant construct.** Mechanisms are grouped according to their most proximal contribution to resistance, brain maintenance, brain reserve, cognitive reserve or resilience. These constructs are analytically distinct but biologically overlapping; individual biological mechanisms may contribute to more than one construct. The predominant construct therefore depends on the biological level and outcome being examined. Then proposed effect of each mechanism and some potential markers/measures of their contribution to the defined construct are also outlined. PET, positron emission tomography; CSF, cerebrospinal fluid; APOE, apolipoprotein E; MRS, magnetic resonance spectroscopy; MRI, magnetic resonance imaging; FDG-PET, fluorodeoxyglucose positron emission tomography; EEG, electroencephalography; TMS, transcranial magnetic stimulation; GABA, γ-aminobutyric acid; EMG, electromyography; SV2A, synaptic vesicle glycoprotein 2A; DMN, default mode network; DTI, diffusion tensor imaging; TSPO, translocator protein; p-tau, phosphorylated tau.

| **Mechanistic domain** | **Biological mechanism/pathway** | **Predominant construct** | **Proposed effect/contribution** | **Potential measures/markers** |
| --- | --- | --- | --- | --- |
| **Pathology accumulation** | Reduced amyloid production and aggregation, or enhanced amyloid clearance | Resistance | Reduced development and burden of pathology | Amyloid-PET; plasma and CSF amyloid-β |
| **Pathology propagation** | Reduced tau seeding, aggregation, or trans-synaptic propagation | Resistance | Slower spread of pathology and downstream brain network injury | Tau-PET; CSF p-tau |
| **Genetic susceptibility** | Protective genetic resilience variants (APOE2, APOE3 Christchurch) | Resilience | Attenuate cognitive consequences of pathology | Genotyping |
| **Epigenetic regulation** | DNA methylation, histone modification and increased expression of neuroplasticity-related genes | Cognitive reserve and brain maintenance | Environmental exposures linked to persistent changes in neurotrophic, synaptic and mitochondrial gene expression | DNA methylation; gene expression profiles (BDNF, EGR1, ARC, MEF2, PPAR-δ, PGC-1α, TFAM etc.) |
| **Epigenetic regulation** | Partial reversal of age-related methylation at MECP2 gene locus due to environmental factors | Brain maintenance | Maintains younger epigenetic profile and may mitigate aspects of biological ageing | DNA methylation; gene expression profiles |
| **Mitochondrial energetics** | Efficient phosphocreatine buffering and preserved mitochondrial complex I activity | Brain reserve | Improve neuronal energetic flexibility, supporting neuronal function during increased cognitive demand and slowing tissue injury | ³¹P-MRS, complex I activity assays; mitochondrial biomarkers; MRI; FDG-PET |
| **Synaptic plasticity** | Preserved long-term potentiation and homeostatic synaptic scaling | Cognitive reserve | Support synaptic plasticity under pathological stress | EEG or TMS plasticity paradigms; synaptic biomarkers; |
| **Calcium homeostasis** | Regulated calcium transfer across synapses, endoplasmic reticulum and mitochondria | Brain maintenance | Prevents calcium overload, oxidative stress, mitochondrial dysfunction and synaptic failure | Calcium imaging |
| **Neurotrophic signalling** | BDNF–TrkB and FNDC5/irisin signalling pathways | Cognitive reserve | Enhances synaptic density, neuronal survival, neurogenesis and activity-dependent plasticity | Plasma and CSF biomarkers |
| **Inhibitory control** | Preserved GABAergic control and excitatory/inhibitory balance | Cognitive reserve | Prevention of network hyperexcitability | EEG; EMG; gamma activity |
| **Structural substrate** | Preservation of brain volume, synaptic density, neuronal number and dendritic complexity | Brain reserve | Greater structural capacity to tolerate pathology before clinical impairment becomes apparent | Structural MRI; SV2A-PET; synaptic biomarkers |
| **Adult hippocampal neurogenesis** | Generation of new granule neurons in adult dentate gyrus | Cognitive reserve | Improve efficiency of processing rather than purely replacing lost neurons | Neurogenesis markers (limited) |
| **Synaptic structure** | Spine morphology e.g. mature mushroom spine density, GluN2B-containing receptors | Brain reserve | Maintains efficient synaptic connectivity and memory despite pathological stress | SV2A-PET; CSF synaptic biomarkers; dendritic spine measures (diameter, post-synaptic density) |
| **Network efficiency and flexibility** | Strength of connections within DMN and capacity for frontoparietal recruitment/redistribution | Cognitive reserve | Enables alternative processing and large-scale compensation despite regional damage | Task-based fMRI; resting-state fMRI |
| **Astrocytic support** | Neurotransmitter recycling, gliotransmitter release, lactate provision and cerebral blood flow regulation | Cognitive reserve | Supports synaptic signalling and network stability through neurotransmitter regulation, metabolic buffering and neurovascular coupling | Plasma and CSF markers; D-serine gliotransmitters; neurovascular imaging; vascular markers |
| **Myelin and axonal integrity** | Oligodendrocytes maintain myelin integrity and metabolically support axons | Brain reserve | Maintains processing speed and network integration | DTI; myelin-sensitive MRI |
| **Microglial regulation** | Secrete anti-inflammatory cytokines and neurotrophic factors | Brain maintenance | Reduce chronic inflammatory injury to maintain function | TSPO-PET; plasma and CSF markers; cytokines; BDNF |
| **Integrated clinical outcome** | Better-than-expected cognitive outcome conditional on burden of pathology | Resilience phenotype | Clinical outcome of the above processes | Residual models or moderation models |
